# MeSH on Demand (product review)

**DOI:** 10.29173/jchla29733

**Published:** 2023-12-01

**Authors:** Angélique Roy

**Affiliations:** Health Sciences Librarian Queen’s University, Kingston, ON, Canada

**Product:** MeSH on Demand

**URL:** https://meshb.nlm.nih.gov/MeSHonDemand

## Product Description

**Fig. 1:**
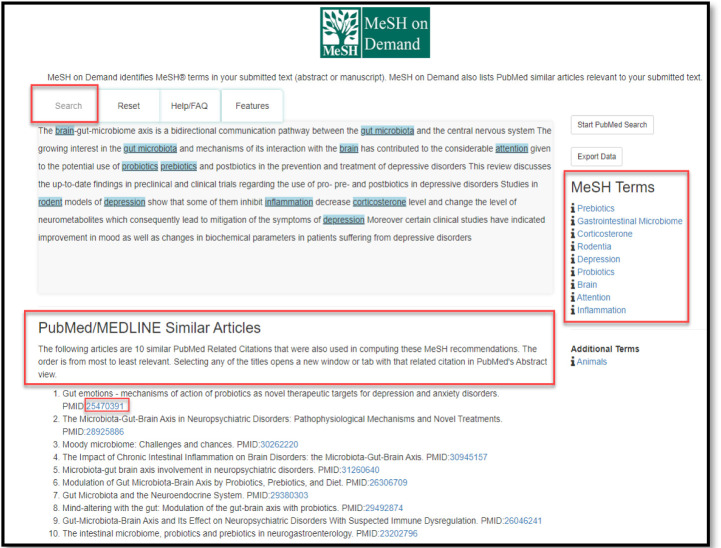
Sample abstract pasted into the query box

MeSH on Demand was launched by the National Library of Medicine (NLM) in 2014 to automatically identify relevant Medical Subject Headings (MeSH) from user-supplied text, such as an article abstract [1]. Text can be pasted or typed into the query box and a search will take an average of 30 seconds to process. Longer text blocks may take upwards of 45 seconds or longer to complete. For example, as seen in Figure 1, copying and pasting an article abstract will generate a list of MeSH Terms appearing on the right side of the query box [2]. The number of MeSH Terms extracted is relative to the size and content of the submitted text. Additionally, a list of ten PubMed/MEDLINE Similar Articles is provided below the submitted text, which are ranked in order of relevancy and linked using the article PubMed ID (PMID).

**Fig. 2:**
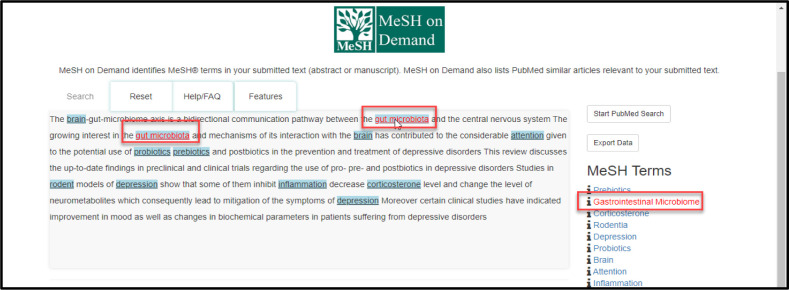
Highlighted text indicating corresponding MeSH Terms

After submitting your text, words or phrases identified as having corresponding MeSH Terms are underlined and highlighted in blue. Hovering the cursor over these words or phrases in the search box will display the terms and their corresponding MeSH Terms to the right in red font (Figure 2).

**Fig. 3:**
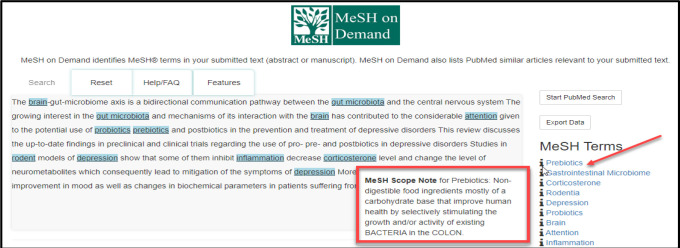
MeSH Scope Note

Running the cursor over the information icon beside a MeSH Term will open a hoverbox with the MeSH Scope Note defining the term (Figure 3).

**Fig. 4:**
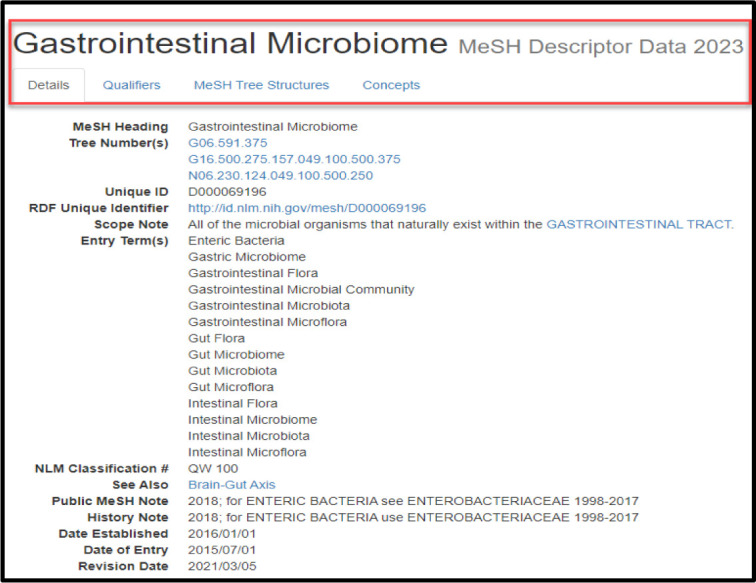
MeSH Browser record opens in a new window

Clicking on a MeSH Term will open a MeSH Browser window displaying the corresponding record which includes Details, Qualifiers, MeSH Tree Structures, and Concepts for further information (Figure 4).

**Fig. 5:**
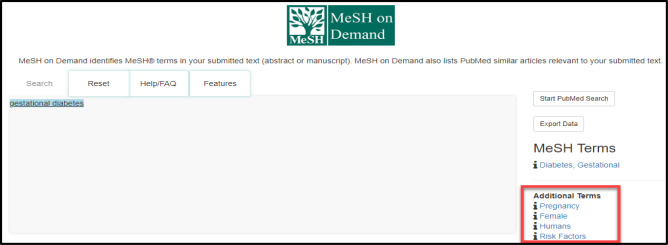
Additional Terms generated from PubMed/MEDLINE Similar Articles

MeSH on Demand also extracts MeSH Terms from the PubMed/MEDLINE related citations based on cooccurrence within the submitted text. These can be found under Additional Terms directly below the list of MeSH Terms (Figure 5). For example, if searching for gestational diabetes on its own, a list of four additional terms is generated given that the condition is specific to pregnancy.

MeSH on Demand uses Natural Language Processing (NLP) and the NLM Medical Text Indexer (MTI) to extract data. MeSH Terms appear in a weighted rank order based on relevance predicted from the processed text. The list of PubMed/MEDLINE Similar Articles are identified based on frequency of common keywords among citations using the National Center for Biotechnology (NCBI)'s PubMed Related Citations algorithm [3]. The MTI, which has been used to assist NLM indexers since 2002 [4], may suggest MeSH Terms that do not appear within the text as the tool does not integrate any human intervention.

## Intended Users

MeSH on Demand was developed in response to feedback that authors found it difficult to identify relevant MeSH Terms for their manuscripts, a requirement when submitting to NLM journals [5]. Given its usefulness in identifying relevant subject headings for submitted text, researchers and librarians conducting literature searches of any type may find the tool valuable in developing a list of MeSH Terms for developing search strategies.

## Special Features

Extracted data can be exported to a text file. After clicking on the Export Data button, a window will open in the same tab where the text file can be saved or printed. Hitting the back button will return an empty search screen so right-clicking on the button and opening a new tab may be preferrable if working from the text file is not ideal.

**Fig. 6:**
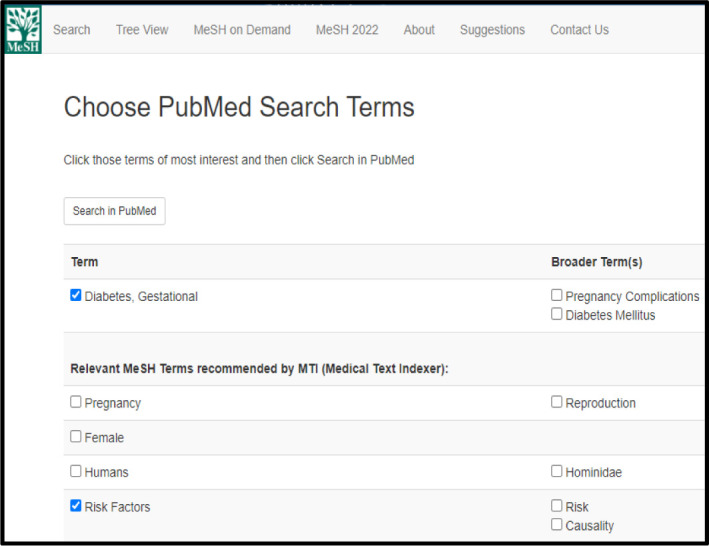
Choose PubMed Search Terms opens in a new window

The Start PubMed Search button, located to the right of the search box and above the MeSH Terms, opens a new window where you can select from the terms that were generated by the MeSH on Demand tool to run a search in PubMed (Figure 6). These also include Broader Term(s) and Relevant MeSH Terms recommended by MTI.

**Fig. 7:**
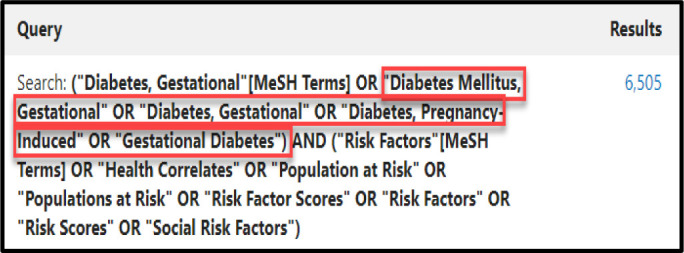
PubMed Advanced Search displaying a search from MeSH on Demand

The PubMed search can then be edited after the results are displayed by adding additional terms and/or revising the Boolean logic. Opening the Advanced Search in PubMed demonstrates that the feature also searches for the Used For terms of the selected MeSH Terms to capture relevant articles from non-MEDLINE journals or those that have not been indexed (Figure 7).

## Platform and Compatibility

MeSH on Demand is web-based and functions well on all major web browsers including Chrome, Firefox, Microsoft Edge, and Safari. It can also be used on a mobile web browser though it is not optimized for this functionality.

## Usability

**Fig. 8:**
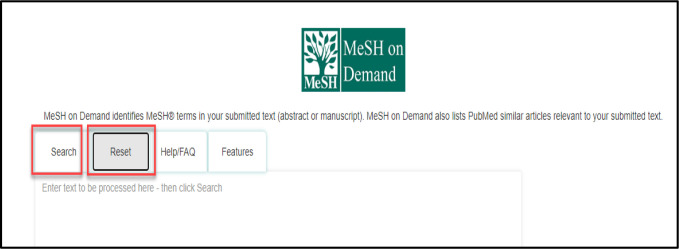
Search and Reset buttons

MeSH on Demand is easy to use and requires minimal steps to retrieve the desired outcome. The display uses a combination of hover boxes and links to explore results. Figure 8 demonstrates that after pasting a search query into the box, the function will only be executed after clicking on Search from this menu. Hitting the enter key will not run the search. New searches can be conducted by clicking on Reset to clear the query box and input new text.

## Strengths

MeSH on Demand is an effective and efficient way to identify relevant subject headings within a sample text and to explore similar articles on a chosen topic. The tool can successfully extract MeSH Terms and PMIDs for upwards of 10,000 characters with spaces but works best in the 7000 character or less range. When approaching the limit, a warning will appear, and resetting will be required if the limit is exceeded.

## Weaknesses

**Fig. 9:**
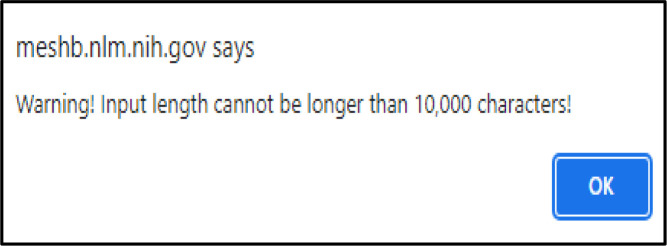
Warning message for text exceeding 10,000 characters

**Fig 10:**
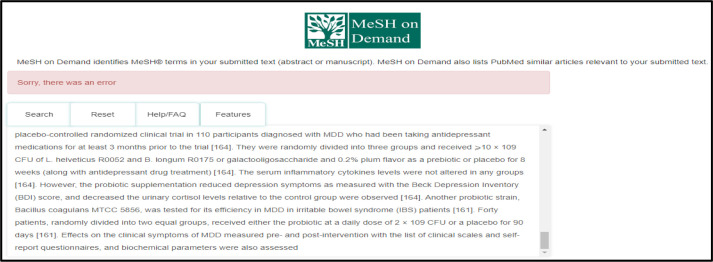
Processing error for text between 9000-10,000 characters

While it is stated that the tool can process up to five pages or 10,000 characters with spaces, inputting this much data causes an error. Five pages is typically in excess of the 10,000-character limit and inputting data between 9000-10,000 characters is met with various warnings (Figure 9) and may result in a processing error (Figure 10).

Currently, MeSH on Demand only processes English text and Latin alphabetic letters. All non-English text and non-alphabetic letters must be translated or converted prior to submitting a search within the tool.

## Comparison with Similar Products

Similar tools that use NLP are available to extract data from PubMed articles or blocks of text.

Yale MeSH Analyzer

The Yale MeSH Analyzer allows for the input of up to 20 PMIDs of relevant articles to analyze contents and create a tabular visualization of MeSH Terms, called a MeSH Analysis Grid. There is also an option to include additional metadata such as abstract, author keywords, and field codes. The grid can be exported to a simple HTML webpage or an Excel spreadsheet.

PubMed PubReMiner

Similarly, in PubMed PubReMiner, searching a set of PMIDs or a free text search that can be processed by PubMed will display relevant subject headings as well as other fields including authors, journal names, substances, and keyword frequency. Searches can be run from PubMed PubReMiner directly back to PubMed. All data is displayed in tables and can be saved as a text file.

Systematic Review Accelerator Word Frequency Analyzer

The Systematic Review Accelerator (SRA) Word Frequency tool serves a similar purpose but does not include MeSH Terms. Keywords are ranked in order of frequency extracted from a user-uploaded file and results can be copied to the clipboard as a basic table.

## Cost

MeSH on Demand is freely available. No account is required.

## Conclusion

MeSH on Demand provides a simple method for determining relevant subject headings from a short block of text. While it does not analyze sets of PMIDs, the PubMed/MEDLINE Similar Articles feature is a helpful way of getting started with a search. Text mining tools such as MeSH on Demand may be particularly useful for discovering relevant MeSH within abstracts and article texts for developing search strategies or to browse additional relevant papers.
